# Thrombosis and antiphospholipid antibodies in Japanese COVID-19: based on propensity score matching

**DOI:** 10.3389/fimmu.2023.1227547

**Published:** 2023-10-16

**Authors:** Seiya Oba, Tadashi Hosoya, Risa Kaneshige, Daisuke Kawata, Taiki Yamaguchi, Takahiro Mitsumura, Sho Shimada, Sho Shibata, Tomoya Tateishi, Ryuji Koike, Shuji Tohda, Akihiro Hirakawa, Nukui Yoko, Yasuhiro Otomo, Junzo Nojima, Yasunari Miyazaki, Shinsuke Yasuda

**Affiliations:** ^1^ Department of Rheumatology, Graduate School of Medical and Dental Sciences, Tokyo Medical and Dental University (TMDU), Tokyo, Japan; ^2^ Department of Laboratory Science, Faculty of Health Science, Yamaguchi University Graduate School of Medicine, Ube, Japan; ^3^ Department of Respiratory Medicine, Graduate School of Medical and Dental Sciences, Tokyo Medical and Dental University (TMDU), Tokyo, Japan; ^4^ Department of Respiratory Medicine, Respiratory Center, Toranomon Hospital, Tokyo, Japan; ^5^ Clinical Laboratory, Tokyo Medical and Dental University (TMDU) Hospital, Tokyo, Japan; ^6^ Department of Clinical Biostatistics, Graduate School of Medical and Dental Sciences, Tokyo Medical and Dental University (TMDU), Tokyo, Japan; ^7^ Department of Infectious Diseases, Division of Comprehensive Patient Care, Medical and Dental Sciences, Graduate School of Medical and Dental Sciences, Tokyo Medical and Dental University (TMDU), Tokyo, Japan; ^8^ Department of Infection Control and Laboratory Medicine, Kyoto Prefectural University of Medicine, Kyoto, Japan; ^9^ Trauma and Acute Critical Care Medical Center, Graduate School of Medical and Dental Sciences, Tokyo Medical and Dental University (TMDU), Tokyo, Japan

**Keywords:** antiphospholipid antibody, beta-2 glycoprotein I, COVID-19, thrombosis, propensity score matching

## Abstract

**Background:**

Thrombosis is a unique complication of coronavirus disease 2019 (COVID-19). Although antiphospholipid antibodies (aPL) are detected in COVID-19 patients, their clinical significance remains elusive. We evaluated the prevalence of aPL and serum concentrations of beta-2 glycoprotein I (β2GPI), a major self-antigen for aPL, in Japanese COVID-19 patients with and without thrombosis.

**Methods:**

This retrospective single-center nested case-control study included 594 hospitalized patients with COVID-19 between January 2020 and August 2021. Thrombotic complications were collected from medical records. Propensity score-matching method (PSM) (1:2 matching including age, sex, severity on admission, and prior history of thrombosis) was performed to compare the prevalence and titer of aPL (anti-cardiolipin (aCL) IgG/IgM, anti-β2GPI IgG/IgM/IgA, and anti-phosphatidylserine/prothrombin antibody (aPS/PT) IgG/IgM) and serum β2GPI concentration. In addition, PSM (1:1 matching including age and sex) was performed to compare the serum β2GPI concentration between COVID-19 patients and healthy donors.

**Results:**

Among the patients, 31 patients with thrombosis and 62 patients without were compared. The prevalence of any aPLs was indifferent regardless of the thrombosis (41.9% in those with thrombosis *vs.* 38.7% in those without, *p* =0.82). The positive rates of individual aPL were as follows: anti-CL IgG (9.7% *vs.* 1.6%, *p* =0.11)/IgM (0% *vs.* 3.2%, *p* =0.55), anti-β2GP1 IgG (22.6% *vs.* 9.7%, *p* =0.12)/IgA (9.7% *vs.* 9.7%, *p* =1.0)/IgM (0% *vs.* 0%, *p* =1.0), and anti-PS/PT IgG (0% *vs.* 1.6%, *p* =1.0)/IgM (12.9% *vs.* 21.0%, *p* =0.41), respectively. The aPL titers were also similar regardless of thrombosis. The levels of β2GPI in COVID-19 patients were lower than those in the healthy donors.

**Conclusion:**

Although aPLs were frequently detected in Japanese COVID-19 patients, their prevalence and titer were irrelevant to thrombotic complications. While COVID-19 patients have lower levels of serum β2GPI than healthy blood donors, β2GPI levels were indifferent regardless of thrombosis. Although most of the titers were below cut-offs, positive correlations were observed among aPLs, suggesting that the immune reactions against aPL antigens were induced by COVID-19. We should focus on the long-term thromboembolic risk and the development of APS in the aPL-positive patients with high titer or multiple aPLs.

## 1 Introduction

COVID-19, caused by infection of the severe acute respiratory syndrome coronavirus 2 (SARS-CoV-2), leads to pneumonia and hypercoagulable state (1–3). Atypical and multiple thromboembolic complications, including arterial, venous, and microvessels, are reported in COVID-19 patients (4–7). These prothrombotic properties are considered as immunothrombosis mediated by enhanced coagulation process and activations of monocytes, neutrophils, and platelets (8). During SARS-CoV-2 infection, acquired immune responses resulted in antibody production against various antigen epitopes (9). Intriguingly, multiple autoantibodies were detected in COVID-19 patients, and several autoantibodies against interferon alpha or neurotransmitters were associated with critically ill conditions (10) or neuropsychiatry symptoms in long-COVID patients (11).

This nature of hypercoagulability in COVID-19 resembles in several aspects with antiphospholipid syndrome (APS), which is characterized by the presence of antiphospholipid antibodies (aPL) and thrombotic complications (12, 13). Initial reports demonstrating positivity for aPL in COVID-19 raised the question that COVID-19 and APS might share similar pathogenic mechanisms, namely, thrombotic microangiopathy. Several reports demonstrated that microvascular injury and thrombosis were observed in both conditions due to multiple mechanisms, including endothelial injury, subsequent platelet or complement activation, and release of neutrophil extracellular traps (14, 15). Initial anecdotal reports implicated the complications of atypical thrombosis during COVID-19 with positive results of aPL (16). Subsequently, a high prevalence of aPLs in critically ill patients with COVID-19 has been reported (17–19). However, the precise pathologic contributions of aPLs in developing COVID-19 thrombosis remain unknown because several factors, such as age and severity, are thought to be potential factors associated with the development of COVID-19 thrombosis and aPL formation (20).

Beta-2 glycoprotein I (β2GPI), the major antigen of aPLs, plays a pivotal role in the coagulation cascade (21). The physiological role of β2GPI remains to be elucidated, but it interacts with negatively-charged phospholipid on the injured endothelial cells surface with its hydrophobic loop on domain V, which results in the negative regulation of coagulation (22). On the other hand, plasmin-cleaved β2GPI binds plasminogen and negatively feedback fibrinolysis (23). Thus, β2GPI plays a role in fine-tuning over coagulation/fibrinolysis system.

Major epitopes for pathological aPL were recognized as domain I of β2GPI (23). Although β2GPI is abundant in circulation, one previous report demonstrated a dramatical decrease in serum levels of β2GPI in COVID-19 patients rather than those in a healthy population (24). Since subclinical thrombolytic activation was often observed regardless of thrombosis, these findings might suggest that the consumption of β2GPI is characteristic of COVID-19. However, it remains unclear whether the development of thrombosis was related to the decreased β2GPI in COVID-19 patients.

This study aimed to investigate the association between the complication of thrombosis and the detection of aPLs in COVID-19 patients, using a propensity score-matching (PSM) approach to minimize the confounding factors. We also aimed to determine β2GPI levels in the COVID-19 patients with thrombosis compared to the healthy controls.

## 2 Materials and methods

### 2.1 Study population

We conducted a nested case-control study of COVID-19 patients admitted at Tokyo Medical and Dental University (TMDU) hospital, a Japanese tertiary emergency hospital in an urban setting. A total of 594 COVID-19 patients consecutively hospitalized between 1 January 2020 and 31 August 2021 were included in this study. The patient’s data were collected until either discharge, transfer to another hospital, or death. The diagnosis of COVID-19 was made by a positive result of a real-time reverse transcription PCR test from nasal swab specimens. Serum samples of COVID-19 patients were obtained and stored at -80°. We excluded COVID-19 cases whose sera were not available. A total of 484 COVID-19 patients were divided into thrombotic patients and non-thrombotic patients (**Table 1**).

**Table 1:** Comparison of the characteristics between the patients with and without thrombosis.

	Non-thrombosis (N=450)	Thrombosis (N=34)	p.value
Baseline characteristics			
Age, median (IQR)	56.50 [45.00, 71.00]	67.50 [60.00, 75.25]	0.00
Male gender, n (%)	310 (68.9)	26 (76.5)	0.44
Body mass index (kg/m2), median (IQR)	23.70 [12.70, 46.21]	24.90 [17.30, 33.43]	0.42
Current smoker, n (%)	75 (16.7)	3 (8.8)	0.33
Severity on admission, n (%)			<0.001
Mild	247 (54.9)	7 (20.6)	
Moderate	110 (24.4)	11 (32.4)	
Severe	93 (20.7)	16 (47.1)	
Comorbidities			
Diabetes	163 (36.2)	20 (58.8)	0.01
Hypertension, n (%)	158 (35.1)	11 (32.4)	0.85
Hyperlipidemia, n (%)	80 (17.8)	4 (11.8)	0.49
History of thrombosis, n (%)	33 (7.3)	6 (17.6)	0.046
History of malignancy, n (%)	59 (13.1)	6 (17.6)	0.436
Laboratory data on admission			
White blood cell count (×10^3^/μL), median (IQR)	5.40 [4.10, 7.20]	7.05 [5.43, 10.07]	0.00
Lymphocyte count (/μL), median (IQR)	978.50 [684.00, 1314.00]	624.40 [448.70, 1120.08]	0.001
Hemoglobin (g/dL), median (IQR)	14.30 [12.90, 15.40]	13.25 [11.75, 14.80]	0.02
Platelet count (×10^4^/μL), median (IQR)	18.75 [15.60, 23.87]	18.10 [13.78, 24.88]	0.63
CRP (mg/dL), median (IQR)	3.84 [0.85, 9.30]	10.48 [6.73, 15.34]	<0.001
LDH (U/L), median (IQR)	289.50 [211.00, 392.00]	419.00 [371.00, 482.00]	<0.001
Ferritin (ng/mL), median (IQR)	427.00 [195.00, 861.50]	750.00 [471.50, 1135.50]	<0.001
Creatinine (mg/dL), median (IQR)	0.84 [0.66, 1.01]	0.86 [0.70, 1.26]	0.10
PT (second), median (IQR)	11.10 [10.40, 11.90]	12.40 [10.95, 14.07]	<0.001
APTT (second), median (IQR)	32.15 [29.50, 35.20]	33.85 [29.22, 37.80]	0.21
D-dimer (μg/mL), median (IQR)	0.70 [0.50, 1.49]	2.18 [1.14, 7.36]	<0.001
Fibrinogen(mg/dL), median (IQR)	473.00 [378.00, 558.00]	578.00 [495.00, 681.00]	<0.001
FDP(μg/mL), median (IQR)	3.20 [2.50, 5.15]	5.95 [3.48, 14.15]	<0.001
Treatment			
Prophylactic anticoagulation dose, n (%)	114 (25.3)	12 (35.3)	0.22
Therapeutic anticoagulation dose, n (%)	37 (8.2)	15 (44.1)	<0.001
Glucocorticoid, n(%)	243 (54.0)	25 (73.5)	0.031
Outcome			
Bleeding, n (%)	8 (1.8)	8 (23.5)	<0.001
Death, n (%)	28 (6.2)	9 (26.5)	<0.001
Transfer, n (%)	102 (22.7)	13 (38.2)	<0.001

*Fisher’s extract test for categorical variables, Mann–Whitney U-test for continuous variables.

IQR, interquartile range; CRP, C-reactive protein; LDH, lactate dehydrogenase; PT,prothrombin time; APTT, activated partial thromboplastin time; FDP, fibrinogen degradation products.

During the admission, 34 patients experienced thrombotic events: 18 were venous thrombosis, and 16 were arterial thrombosis. No cooccurrence of arterial and venous thrombosis was observed. In most cases, the detailed patterns of thrombotic events were described previously (20). Sequential evaluation of respiratory status revealed that thrombosis occurred in 7 cases during exacerbation and 9 cases during improvement. The value of D-dimer elevated from several days before thrombosis was diagnosed (**Supplementary Figure 1**). Our study design complied with the Declaration of Helsinki and the Strengthening the Reporting of Observational Studies in Epidemiology (STROBE) reporting guideline (25). The ethics committees of TMDU approved this study as G2020-034.

### 2.2 Control population

To compare the levels of β2GPI in COVID-19 patients with a healthy population, sera from healthy blood donors (n=80: age range 37-65) collected pre-pandemic period were measured subsequently. Blood donors had no history of thrombotic events or symptoms at the time of blood donation. To define the healthy donors, we adopted the following criteria for exclusion: 1. body mass index (BMI) ≥ 28kg/m^2^, 2. consumption of ethanol ≥ 75g/day, 3. ≥20 cigarettes/day, 4. under drug therapy, and 5. pregnancy ≤1 year after childbirth, as described previously (26).

### 2.3 Data collection

We collected clinical data from electronic medical records, including demographic information, comorbidities, type of thrombosis, laboratory data, treatment, and outcomes as described previously (20). Severity was defined as mild for patients who do not need supplemental oxygen, moderate for patients who need supplemental oxygen of less than 4 L/min, and severe for patients who need supplemental oxygen of more than 5 L/min or intubation. As an anticoagulation therapy, a therapeutic dose of unfractionated heparin was defined as the dose determined in reference to the activated partial thromboplastin time (APTT), while a prophylactic dose was defined as a fixed dose of unfractionated heparin (equal or less than 10,000U/day) regardless of the APTT.

### 2.4 Outcomes

Our primary outcome was to compare the prevalence of aPLs among COVID-19 patients with and without thrombosis. Secondary outcomes included comparing the level of β2GPI between COVID-19 patients and healthy donors, and the associations with thrombotic markers and aPL.

### 2.5 Propensity score matching methods

We used propensity score matching to ensure a balanced covariates distribution between patients with and without thrombosis. Propensity scores were calculated using a multivariate logistic regression model with several potential confounding factors identified based on previous reports and clinical knowledge, namely, sex, the severity on admission, and prior history of thrombosis (20). Propensity scores were matched using a 1:2 protocol without replacement. The caliper width was 0.2 logit of the standard deviation of estimated propensity scores (27). Regarding the thirty-four thrombotic cases, each case was matched with two non-thrombotic cases. The corresponding propensity scores indicated an appropriate balance of covariates.

We also used PSM to balance the baseline characteristics of COVID-19 patients and healthy controls. Potential confounders were identified, namely age and sex. The matching quality was assessed using standardized mean difference (SMD) (28). Covariates with SMD < 0.25 are considered moderately balanced (29), and those with SMD < 0.1 are considered highly balanced (30).

**Figure 1:**
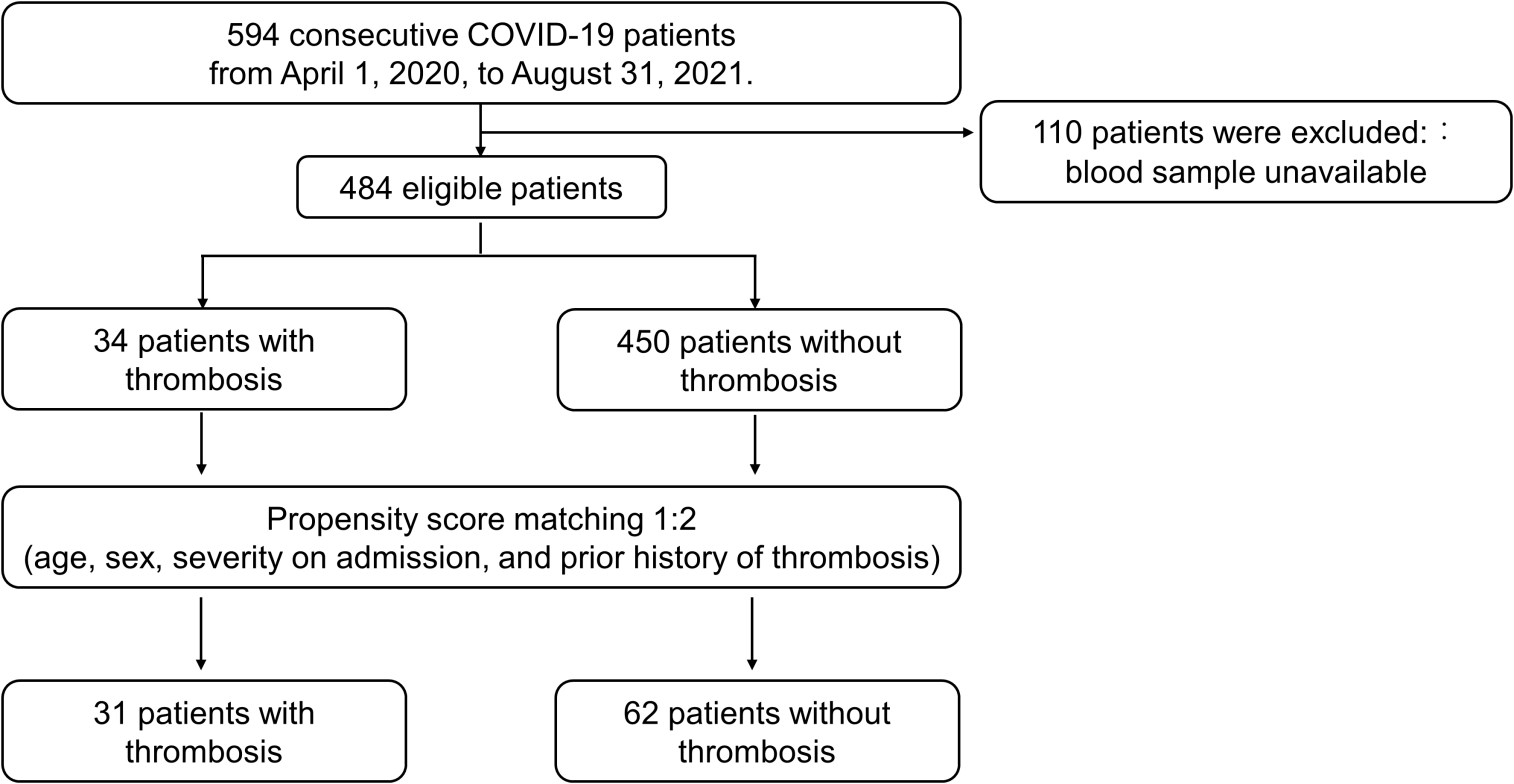
Flowchart for the Propensity score matching analysis. The diagram presents included and excluded patients before and after propensity score matching.

Finally, 31 patients with thrombosis and 62 patients without thrombosis were compared for the prevalence of aPL and β2GPI level (**Figure 1**); the differences of baseline variables were attenuated in the propensity score-matched cohort compared to the unmatched cohort (**Table 2**). No APS patients were found in our study cohort.

**Table 2:** COVID-19 patients’ characteristics before and after propensity score matching.

	Before PSM			After PSM		
	Non-thrombosis (N=450)	Thrombosis (N=34)	SMD	Non-thrombosis (N=62)	Thrombosis (N=31)	SMD
Baseline characteristics						
Age, median (IQR)	56.50 [45.00, 71.00]	67.50 [60.00, 75.25]	0.631	68.50 [57.3, 74.8]	68.00 [58.00, 74.50]	0.073
Male gender, n (%)	310 (68.9)	26 (76.5)	0.171	50 (80.6)	25 (80.6)	<0.001
Body mass index (kg/m2), median (IQR)	23.70 [12.70, 46.21]	24.90 [17.30, 33.43]	0.046	23.75 [21.5, 26.5]	24.70 [21.60, 27.10]	0.25
Current smoker, n (%)	75 (16.7)	3 (8.8)	0.24	9 (15.8)	3 (11.1)	0.14
Severity on admission, n (%)			0.79			<0.001
Mild	247 (54.9)	7 (20.6)		14 (22.6)	7 (22.6)	
Moderate	110 (24.4)	11 (32.4)		22 (35.5)	11 (35.5)	
Severe	93 (20.7)	16 (47.1)		26 (41.9)	13 (41.9)	
Comorbidities						
Diabetes, n (%)	163 (36.2)	20 (58.8)	0.465	35 (56.5)	17 (54.8)	0.032
Hypertension, n (%)	158 (35.1)	11 (32.4)	0.058	25 (40.3)	8 (25.8)	0.312
Hyperlipidemia, n (%)	80 (17.8)	4 (11.8)	0.17	9 (14.5)	3 (9.7)	0.15
History of thrombosis, n (%)	33 (7.3)	6 (17.6)	0.316	8 (12.9)	4 (12.9)	<0.001
History of malignancy, n (%)	59 (13.1)	6 (17.6)	0.126	13 (21.0)	6 (19.4)	0.04
Treatment						
Prophylactic anticoagulation dose, n (%)	114 (25.3)	12 (35.3)	0.218	28 (45.2)	11 (35.5)	0.198
Therapeutic anticoagulation dose, n (%)	37 (8.2)	15 (44.1)	0.894	5 (8.1)	14 (45.2)	0.925
Glucocorticoid, n (%)	243 (54.0)	25 (73.5)	0.415	46 (74.2)	23 (74.2)	<0.001

PSM, propensity score matching; SMD, standardized mean difference; IQR, interquartile range.

### 2.6 Measurements of biomarkers

All the matched patients were evaluated for aPL. An antigen-coated–beads automatized assay (LSI Medience Corporation) measured the classic aPL, anti-CL IgG/IgM and aβ2GPI IgG/IgM. The cutoffs shown by the supplier were 20 U/ml. The non-criteria aPL, anti-phosphatidylserine/prothrombin antibody (aPS/PT) IgG/IgM (INOVA Diagnostics) and aβ2GPI IgA (IBL international GmbH), were analyzed by enzyme-linked immunosorbent assay (ELISA). Because of the limitation of commercial availability, we could only analyze the IgA subclass of β2GPI but not of CL and PS/PT. The cutoffs were 30 U/ml and 12 U/ml for aPS/PT IgG/IgM and aβ2GPI IgA, respectively. These values corresponded for each method to the 99th percentile of a healthy population as provided by the supplier. Serum levels of β2GPI were quantified using Apolipoprotein H (APOH) ELISA Kit (Aviscera Bioscience).

The serum levels of P-selectin (R&D systems) and Plasminogen activator inhibitor type 1(PAI-1) (Proteintech) were evaluated by ELISA. All assays were performed according to the manufacturer’s protocols and interpreted using the manufacturers’ cutoff values.

### 2.7 Statistical analysis

Continuous variables were shown as the median and interquartile range (IQR). Categorical variables were shown as absolute numbers and percentages. Mann-Whitney test was used for continuous variables. Categorical variables were compared with Fisher’s exact test. One-way ANOVA followed by Tukey-Kramer *post hoc* test was performed to analyze the titers of aPLs and time points of sampling days post onset. A p-value < 0.05 was considered statistically significant. The Spearman correlation coefficient was used to determine the correlation among the titers of aPLs, the serum levels of β2GPI and the several biomarkers. Considering the multiple testing, we used adjusted p-value (p<0.0018) following the Bonferroni correction in **Supplementary Figure 4**. All the statistical analyses were conducted using GraphPad Prism software version 8.0 (GraphPad Software), or EZR software version 1.54, free software for using R on a graphical user interface (31).

## 3 Results

### 3.1 Prevalence and concentration of aPL in COVID-19 patients with and without thrombosis

Overall, 39.0% of patients had at least one positive aPL. The prevalence of any aPL was comparable in patients with and without thrombosis [41.9% *vs.*38.7%, *p* =0.82 (**Table 3**)]. No significant differences were found in the prevalences of individual classical and non-criteria aPLs in the two groups. Regarding the prevalence of aPL according to the type of thrombosis, no differences were observed in the types of thrombosis (**Supplementary Table 1**).

**Table 3:** Prevalence of aPL in COVID-19 patients with and without thrombosis.

	All patients (n=93)	Non-thrombosis (n=62)	Thrombosis (n=31)	*p.* value
Any aPL (%)	37 (39.0)	24 (38.7)	13 (41.9)	0.82
Classical aPL (%)	18 (19.4)	9 (14.5)	9 (29.0)	0.11
aCL IgG (%)	4 (4.3)	1 (1.6)	3 (9.7)	0.11
aCL IgM (%)	2 (2.2)	2 (3.2)	0 (0.0)	0.55
aβ2GPI IgG (%)	13 (14)	6 (9.7)	7 (22.6)	0.12
aβ2GPI IgM (%)	0 (0)	0 (0.0)	0 (0.0)	NA
Non-criteria aPL (%)	25 (26.3)	18 (29.0)	7 (22.6)	0.62
aPS/PT IgG (%)	1 (1.1)	1 (1.6)	0 (0.0)	1
aPS/PT IgM (%)	17 (18.3)	13 (21.0)	4 (12.9)	0.41
aβ2GPI IgA (%)	9 (9.5)	6 (9.7)	3 (9.7)	1

aPL, antiphospholipid antibody; aCL, anti-cardiolipin; β2GPI, beta-2 glycoprotein I; aPS/PT; anti-phosphatidylserine/prothrombin. NA; Not applicable.

**Figure 2:**
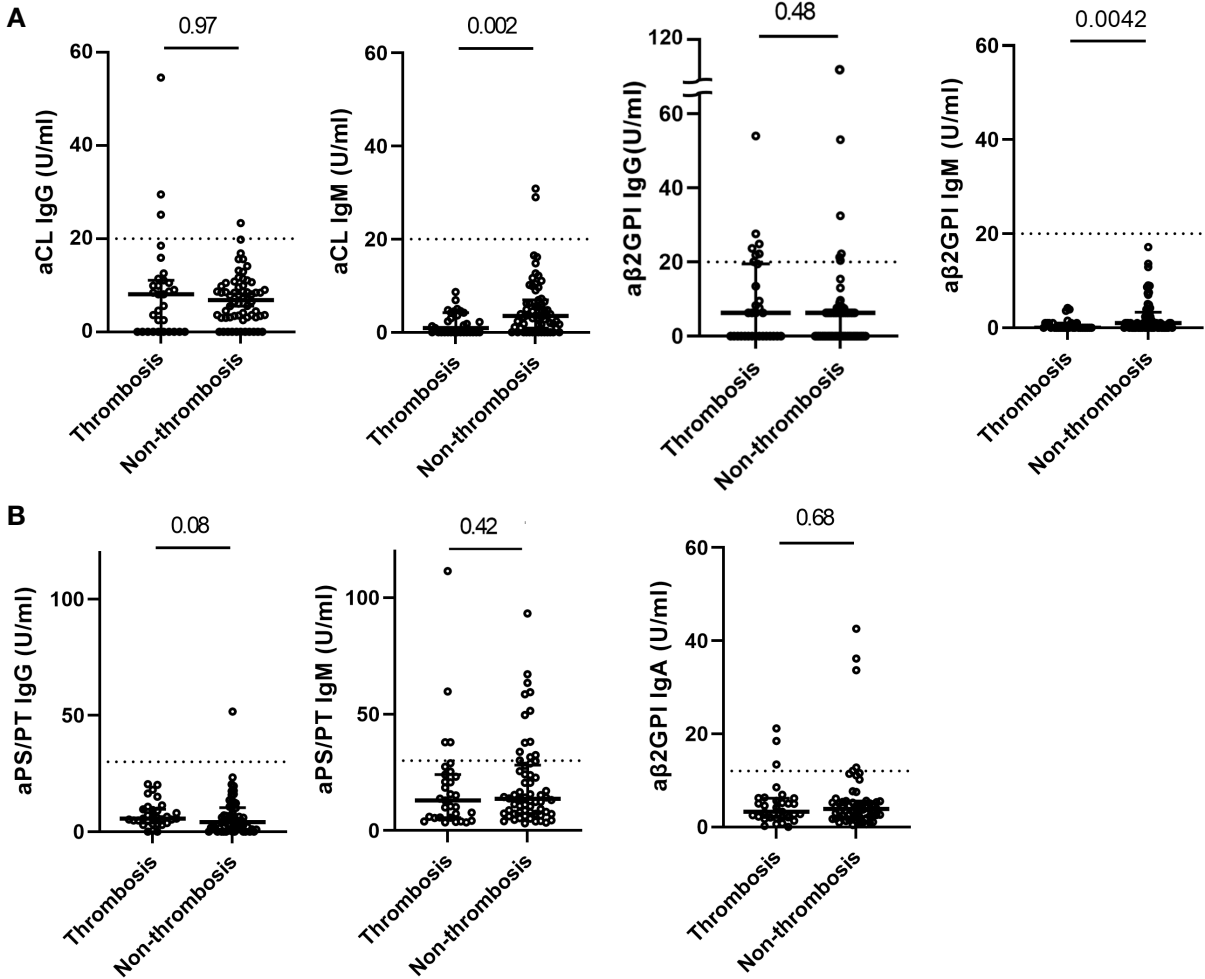
Distribution of aPL in COVID-19 patients with and without thrombosis. Titers of classic aPL **(A)** (anti-cardiolipin (aCL) IgG/IgM, anti-beta-2glycoprotein I (aβ2GPI) IgG/IgM) detected by a chemiluminescence analyzer, and titers of non-criteria aPL **(B)** (aβ2GPI IgA and anti-phosphatidylserine/prothrombin (aPS/PT) IgG/IgM) detected by ELISA in COVID-19 patients with (n=31) and without thrombosis (n=62). Values are expressed as median levels [first and third quartile]. Broken lines represent the manufacturer’s cutoff for positivity (20 U/ml for classic aPL, 30 U/ml for aPS/PT IgG/IgM, and 12 U/ml for aβ2GPI IgA). Groups were analyzed by Mann-Whitney U-test.

The titer of aPL was almost similar regardless of thrombosis complicated during COVID-19 or prior history of thrombotic events, except for aCL IgM (0.9 U/ml versus 3.3 U/ml, *p* =0.002) and aβ2GPI IgM (0 U/ml versus 1.0 U/ml, *p*=0.0042) (**Figure 2**, **Supplementary Figure 2**). Most positive aPL determinations were at low titers regardless of thrombosis, compared to the APS patients.

When we divided the timepoint of sampling days post onset (DPO), anti-β2GPI IgG antibody levels were higher at the latest timepoint (**Supplementary Figures 3**). Interestingly, among IgM aPLs of CL, β2GPI, and PS/PT had correlations (**Supplementary Figure 4**).

### 3.3 Comparison of serum β2GPI concentration in COVID-19 patients and healthy controls

**Figure 3:**
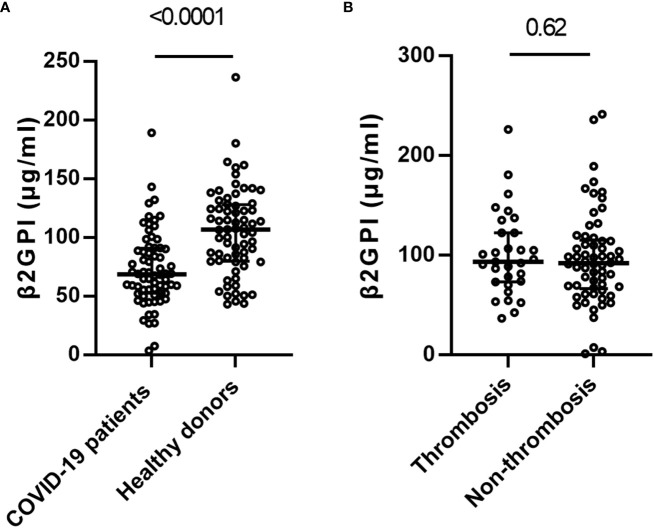
Distribution of beta-2 glycoprotein I (β2GPI) in COVID-19 patients and healthy controls. **(A)** β2-glycoprotein-I(β2GPI) levels in the patients with COVID-19 (n=68) versus the healthy blood donors (n=68). **(B)** β2GPI levels in the patients with COVID-19 patients with (n=31) and without thrombosis (n=62). Values are expressed as median levels [first and third quartile]. Groups were analyzed by Mann-Whitney U-test.

We compared β2GPI levels between COVID-19 patients and healthy blood donors. We found differences between COVID-19 patients(n=484) and healthy donors(n=80) of median age (57.5 *vs.* 53.0 years) and male gender (69.4% *vs.* 77.5%). To minimize this bias, PSM was performed, and baseline characteristics were balanced (median age:54.5 *vs.* 55 years, SMD=0.069, male gender:75% *vs.* 78%, SMD=0.012). After PSM, those 68 matched pairs of COVID-19 patients and healthy blood donors were compared. COVID-19 patients had significantly lower levels of β2GPI concentrations than healthy donors (68.7[IQR:52.6-90.5] ug/ml *vs.* 106.8 [IQR:80.2-127.3]ug/ml, *p <*0.001) (**Figure 3A**), consistent with the previous report, whereas no significant difference was observed in β2GPI concentrations between healthy donors and COVID-19 thrombosis patients (data not shown).

In the COVID-19 patients, no significant difference was identified in the level of β2GPI between thrombosis and non-thrombosis COVID-19 patients (92.0 ug/ml [IQR: 67.6-114.4] *vs.* 93.7 ug/ml [IQR: 73.8, 122.7], *p* =0.62) (**Figure 3B**).

### 3.4 Relationship of serum levels of β2GPI and coagulation markers

Since β2GPI was an intrinsic negative regulator of coagulation and the autoantigen of APLs, we hypothesized that the consumption of β2GPI might reflect the clinical and subclinical activation coagulation/fibrinolysis system. As biomarkers with hypercoagulation, we measured PAI-1, a marker of endothelial dysfunction, and P-selectin, a marker of platelet activation, in addition to the routine biological parameters (CRP, D-dimer, and ferritin). We then performed a comprehensive analysis of the relationship between serum levels of β2GPI and these biomarkers (**Supplementary Table 2**). β2GPI levels were not significantly associated with any of the biomarkers.

## 4 Discussion

This study investigated the prevalence of aPL in COVID-19 patients with thrombosis compared to those without thrombosis, adjusting for patient background by propensity score matching. The results showed a high prevalence of aPL at around 40% in our COVID-19 patients, with no difference in the prevalence in the two groups. Likewise, the titer of aPL in COVID-19 patients with thrombosis was similar to that in patients without thrombosis. Noteworthy, the levels of β2GPI in COVID-19 patients were lower than in the healthy population.

A few previous studies have reported contradicting results on the prevalence of aPL in COVID-19-associated thrombosis, partly because of differences in patients’ backgrounds and the type of aPL measured (13, 32, 33). Some retrospective cohort studies have reported a higher prevalence of aPL in critically ill COVID-19 patients (34) than in non-severe patients (13, 18). Therefore, confounding factors, including severity, should be adjusted to compare the prevalence of aPL in thrombosed and non-thrombosed cases of COVID-19. Propensity score matching can effectively balance the differences in groups and reduce the effects of confounding (29). Our propensity-matched comparison showed no significant adjusted differences in the prevalence of aPL regardless of thrombosis in COVID-19.

The overall aPL positivity rate was generally consistent with previous studies (17–19, 34–38) (**Supplementary Table 3**). In a meta-analysis, the pooled prevalence of one or more aPL (IgG or IgM isotypes of aCL, aβ2GPI, aPS/PT, or Lupus Anticoagulant) was 46.8% (39). In a cohort of 172 COVID-19 hospitalized patients, the most frequent aPL was aPS/PT IgG (24%)), followed by aCL IgM (23%) and aPS/PT IgM (18%), respectively (35). The prevalence of aPL in healthy donors has been reported to be around 1-5% (40). Regarding aPL subtypes in our cases, 21% were most frequently positive for aβ2GPI IgG, while aCL IgM and aβ2GPI IgM were not detected in any of the patients, indicating that the prevalence of aPL in COVID-19 was high relative to the general population, yet the clinical relevance remains unsolved.

Remarkably, only a few studies have evaluated the aPL titers and specificity in detail. Zuo et al. reported that the prevalence of aPL in COVID-19 patients was 52% using the manufacturer’s threshold, although the percentage decreased to 30% if a more stringent cutoff point (≥ 40 ELISA-specific units) were applied (35). In another study, the median levels of aCL IgG/IgM and aβ2GPI IgG/IgM in COVID-19 patients were lower than in APS (15/4 GPL/MPL unit versus 65/6.2 GPL/MPL unit) (41). Furthermore, they focused on the antigen specificity of COVID-19 aPL compared to APS antibodies. While the medium or high aPL titers against domain I specificity were associated with thrombosis in APS, non-pathogenic antibodies with lower affinity against β2GPI or that recognize other epitopes could be detected in COVID-19. Similarly, Trahtemberg U et al. revealed the prevalence of aCL IgG increased during admission in critically ill patients regardless of COVID-19, whereas aβ2GPI IgG against domain I was detected in none of the patients (37).

Systematic reviews reported that low titer and transient aPLs were detected in various viral infections, not exclusively SARS-CoV-2 (19). In our study, although the values were below the cutoffs for the diagnosis of APS, several correlations were observed among the aPLs and high titer of aβ2GPI IgG was detected in the samples collected at the latest timepoint, suggesting the immune reaction against aPL antigens. Therefore, we considered that the detected aPLs were low titer, non-disease-causing, and transient in most cases of COVID-19. However, environmental factors, including virus or bacterial infection, vaccination, and a part of drugs, might trigger the pathogenic aPLs and lead to the development of true APS in genetically susceptible cases (42). Indeed, Mendel A et al. revealed that the COVID-19 patients with high titers of APLs were associated with thromboembolic event (43), as demonstrated in the patients with APS (44). We should focus on the long-term thromboembolic risk and the development of APS in aPL-positive patients with high titer or multiple aPLs.

Many biomarkers have been investigated for the diagnosis and clinical outcome of COVID-19. In particular, as biomarkers reflecting the pathogenesis of immunothrombosis (45), elevated GAS6 and osteopontin have been noted for their prognostic parameter in COVID-19 (46, 47). Decreased β2GPI levels may also be useful as a unique biomarker in COVID-19. As expected, the levels of β2GPI in COVID-19 patients were lower than those in healthy control. Several studies have reported that the level of β2GPI was lower in severe infection due to higher consumption (24, 48). β2GPI inhibits procoagulant factors (49) and interacts with apoptotic cells (50). β2GPI, which interacts with negatively charged phospholipid expressed in the surface membrane through the positively charged domain V, promotes its incorporation and degradation by macrophages via scavenger receptors (51). Likewise, domain V might bind through negatively charged SARS-CoV-2 and be consumed. Low levels of β2GPI would lead to dysregulation of coagulation and platelet aggregation, thus could be a possible mechanism of thrombus formation (24).

Our study has several limitations. First, the matched cohort was small due to a single center. Therefore, our results need to be verified in a larger cohort before being widely applied. Second, the lupus anticoagulant test was not performed because of the need for access to fresh plasma samples and the high proportion of anticoagulation therapy in our patients. Third, our results lacked information on whether the aPL was persistent or generated as a result of class switch. Since sequential sample collection was not performed, aPL and β2GPI were measured at a single time point. Fourth, we could not utilize genetic data considering the genetic susceptibility of APS or serum levels of β2GPI. Fifth, this study’s propensity score matching results are generalizable only to those in the propensity score range included in the paired analysis. Propensity score methods can reduce bias in causal estimates due to observed differences between two comparable groups. However, it can be subject to biases from unobserved differences (52).

## 5 Conclusion

In summary, we performed a propensity-matched analysis to evaluate the association of thrombotic events and the prevalence of aPL or serum concentrations of β2GPI. APL determinations in our study were unrelated to thrombotic events, even though we analyzed them with detailed clinical information. Additionally, we confirmed significantly lower levels of serum β2GPI in COVID-19 patients than in healthy control. Further studies are required to elucidate the pathogenic role of aPL and its antigen in the clinical manifestations of COVID-19.

## Data availability statement

The raw data supporting the conclusions of this article will be made available by the authors, without undue reservation.

## Ethics statement

The studies involving humans were approved by The ethics committees of Tokyo Medical and Dental University. The studies were conducted in accordance with the local legislation and institutional requirements. Written informed consent for participation was not required from the participants or the participants’ legal guardians/next of kin because it was not required for this study in accordance with the national legislation and the institutional requirements.

## Author contributions

SO, TH, and SY designed the experiments. SO, RKa, and DK performed the experiments. SO, TH, RKa, TY, RKo, AH, and SY analyzed data. SO, DK, TY, TM, SShim, SShib, TT, ST, YN, YO, YM collected the patient information. SO, TH and SY wrote the paper. RKo, JN and SY supervised the manuscript

## Supplementary material

Supplementary Figure 1 Violin plot showed daily variation of D-dimer levels for one week before the onset of thrombosis.

Supplementary Figure 2 Distribution of aPL in COVID-19 patients with and without prior history of thrombotic events. Titers of classic aPL **(A)** (anti-cardiolipin (aCL) IgG/IgM, anti-beta-2glycoprotein I (aβ2GPI) IgG/IgM) detected by a chemiluminescence analyzer, and titers of non-criteria aPL **(B)** (aβ2GPI IgA and anti-phosphatidylserine/prothrombin (aPS/PT) IgG/IgM) detected by ELISA in COVID-19 patients with (n=12) and without prior history of thrombotic events (n=82). Values are expressed as median levels [first and third quartile]. Broken lines represent the manufacturer’s cutoff for positivity (20 U/ml for classic aPL, 30 U/ml for aPS/PT IgG/IgM, and 12 U/ml for aβ2GPI IgA). Groups were analyzed by Mann-Whitney U-test.

Supplementary Figure 3 The relation of the aPL titers and sampling time points post onset. The samples were categorized into four groups based on the collected days of post onset (DPO). The groups consisted of the samples collected within a week, between 7-14 days, between 15-21 days, and more than 22 days, and the numbers of samples were 21, 44, 18, and 10, respectively. Values are expressed as box-and-whisker plot. Groups were analyzed by one-way ANOVA followed by Tukey-Kramer *post hoc* test. We showed statistically significant adjusted p-values (overall alpha = 0.05).

Supplementary Figure 4 Correlations among classical aPLs, non-criteria aPLs, and serum levels of β2GPI. Considering the multiple comparisons, we used p< 0.0018(0.05/28) as statistically significant and added asterisks (*).
