# Supplementary material for: Thrombosis and antiphospholipid antibodies in Japanese COVID-19: based on propensity score matching
Source: Front Immunol. 2023 Oct 16;14:1227547. doi: 10.3389/fimmu.2023.1227547 (PMC10614020; doi:10.3389/fimmu.2023.1227547)
Supplement: Supplementary file 1 [file DataSheet_1.docx]

Supplementary Material

Thrombosis and Antiphospholipid Antibodies in Japanese COVID-19: Based on Propensity score matching

**Seiya Oba^1^, Tadashi Hosoya^1^*, Risa Kaneshige^2^, Daisuke Kawata^1^, Taiki Yamaguchi^1^, Takahiro Mitsumura^3.4^ , Sho Shimada^3^, Sho Shibata^3^, Tomoya Tateishi^3^ , Shuji Tohda^5^, Akihiro Hirakawa^6^ , Yoko Nukui^7.8^, Yasuhiro Otomo^9^, Junzo Nojima^2^, Yasunari Miyazaki^3^, Shinsuke Yasuda^1^***

*** Correspondence:**

Tadashi Hosoya. E-mail: hosorheu@tmd.ac.jp

Shinsuke Yasuda. E-mail: syasuda.rheu@tmd.ac.jp

Supplementary Table 1. The association between the type of thrombosis and aPL.

|  | Pulmonary thromboembolism  (n=15) | Deep vein thrombosis  (n=7) | Acute coronary syndrome  (n=8) | Cerebral infarction  (n=5) | Intracardiac thrombosis  (n=2) | Splenic infarction  (n=3) | Aortic thrombosis  (n=2) | Peripheral arterial obstruction  (n=1) | Renal infarction  (n=1) |
| --- | --- | --- | --- | --- | --- | --- | --- | --- | --- |
| Any aPL | 47% | 57% | 50% | 20% | 50% | 0% | 0% | 0% | 0% |
| Classical aPL | 33% | 29% | 25% | 20% | 0% | 0% | 0% | 0% | 0% |
| aCL IgG | 20% | 0% | 0% | 0% | 0% | 0% | 0% | 0% | 0% |
| aCL IgM | 0% | 0% | 0% | 0% | 0% | 0% | 0% | 0% | 0% |
| aβ2GPI IgG | 20% | 29% | 25% | 20% | 0% | 0% | 0% | 0% | 0% |
| aβ2GPI IgM | 0% | 0% | 0% | 0% | 0% | 0% | 0% | 0% | 0% |
| Non-criteria aPL | 20% | 43% | 25% | 20% | 50% | 0% | 0% | 0% | 0% |
| aPS/PT IgG | 0% | 0% | 0% | 0% | 0% | 0% | 0% | 0% | 0% |
| aPS/PT IgM | 13% | 29% | 13% | 0% | 50% | 0% | 0% | 0% | 0% |
| aβ2GPI IgA | 0% | 14% | 13% | 20% | 0% | 0% | 0% | 0% | 0% |

aPL,antiphospholipid antibody; aCL,anti-cardiolipin; β2GPI, beta-2 glycoprotein I; aPS/PT; anti-phosphatidylserine/prothrombin.

Supplementary Table 2. Correlation of β2GPI with clinical and laboratory variables in patients with COVID-19.

|  | β2GPI concentration | | |
| --- | --- | --- | --- |
|  | γ | 95% CI | p.value |
| Age | -0.0131 | -0.2220 to 0.1970 | 0.9008 |
| Body mass index | 0.07301 | -0.1435 to 0.2828 | 0.4965 |
| White blood cell count | -0.05862 | -0.2649 to 0.1528 | 0.5767 |
| Platelet | -0.08729 | -0.2915 to 0.1245 | 0.4054 |
| CRP on admission | 0.01316 | -0.1969 to 0.2221 | 0.9004 |
| D-dimer on admission | 0.1934 | -0.01681 to 0.3873 | 0.0632 |
| Peak CRP | 0.04723 | -0.1639 to 0.2543 | 0.653 |
| Peak D-dimer | 0.1216 | -0.09027 to 0.3229 | 0.2457 |
| Ferritin | -0.2175 | -0.4409 to 0.03115 | 0.077 |
| P-selectin | 0.09487 | -0.1170 to 0.2985 | 0.3657 |
| PAI-1 | 0.06791 | -0.1437 to 0.2736 | 0.5178 |

β2GPI, beta-2 glycoprotein I; CI, Confidential Interval; CRP, C reactive protein; PAI-1, Plasminogen activator inhibitor type 1.

Supplementary Table 3. Comparison with the similar articles

|  | **Patient population** | **thrombosis** | **aPL(%)** | **aCL IgG (%)** | **aCL IgM (%)** | **aβ2GPI IgG (%)** | **aβ2GPI IgM(%)** | **aPS/PT IgG(%)** | **aPS/PT IgM(%)** | **aβ2GPI IgA(%)** | **Type of test** | **Cut-off value (manufacturer's cut off)** |
| --- | --- | --- | --- | --- | --- | --- | --- | --- | --- | --- | --- | --- |
| **Our study (n=93)** | **General ward and ICU** | **31** | **37** | **4.3** | **2.2** | **14** | **0** | **1.1** | **18** | **6.5** | **CLIA** | **20 units for aCL IgG/IgM, and aβ2GPI IgG/IgM** |
|  |  |  |  |  |  |  |  |  |  |  | **ELISA** | **30 units for aPS/PT IgG/IgM and 12 units for aβ2GPI IgA** |
| Zuo(35) (n=172) | General ward and ICU | N/D | 52 | 4.7 | 23 | 2.9 | 5.2 | 24 | 18 | 4.1 | ELISA | 20 units for aCL IgG/IgM/IgA and aβ2GPI IgG/IgM/IgA |
|  |  |  |  |  |  |  |  |  |  |  |  | 30 units for aPS/PT IgG and IgM |
| Gendron(17) (n=149) | General ward and ICU | 40 | 47 | 5.8 | 1.3 | 3.2 | 1.9 | 0 | 4.5 | 1.3 | CLIA | 20 units for aCL IgG/IgM/IgA and aβ2GPI IgG/IgM/IgA |
|  |  |  |  |  |  |  |  |  |  |  | ELISA | 30 Units for aPS/PT IgG and IgM |
| Xiao(18) (n=79) | General ward | 25 | 47 | 5.1 | 2.5 | 15 | 1.3 | 0 | 8.9 | 24 | CLIA | 20 units for aCL IgG/IgM/IgA and aβ2GPI IgG/IgM/IgA |
|  |  |  |  |  |  |  |  |  |  |  | ELISA | 30 Units for aPS/PT IgG/IgM |
| Previtali(34)(n=35) | General ward | 10 | 8.6 | 4 | 5.7 | 0 | 0 | 4 | 5.7 | 0 | CLIA | 20 units for aCL IgG/IgM/IgA and aβ2GPI IgG/IgM/IgA |
|  |  |  |  |  |  |  |  |  |  |  |  |  |
|  |  |  |  |  |  |  |  |  |  |  | ELISA | 30 Units for aPS/PT IgG/IgM |
|  |  |  |  |  |  |  |  |  |  |  |  |  |
| Cristiano (36) (n=92) | General ward | ND | ND | 3.3 | 1.1 | 0 | 2.2 | 2.2 | 3.3 | ND | CLIA | 20 units for aCL IgG/IgM and aβ2GPI IgG/IgM |
| Trahtemberg(37) (n=22) | ICU | ND | ND | 59 | 32 | 0 | 0 | 0 | 4.5 | ND | ELISA | 20 units |
| Amezcua-Guerra(38)(n=21) | ICU | 2 | 57 | 8.3 | 12.5 | 4.2 | 0 | 8.3 | 17 | 0 | ND | ND |
|  |  |  |  |  |  |  |  |  |  |  |  |  |
| Devreese (19) (n=31) | ICU | 12 | 74 | 20 | 3.2 | 9.7 | 3.2 | 9.7 | 13 | 9.7 | CLIA | 20 units for aCL IgG/IgM/IgA and aβ2GPI IgG/IgM/IgA |
|  |  |  |  |  |  |  |  |  |  |  | ELISA | 30 units for aPS/PT IgG/IgM |

Our study is written in bold. aPL,antiphospholipid antibody; aCL, anti-cardiolipin; β2GPI, beta-2 glycoprotein I; aPS/PT; anti-phosphatidylserine/prothrombin; ICU, Intensive Care Unit; ND, Non data; CU, Chemiluminescent Units; CLIA, chemiluminescence immunoassay; ELISA, Enzyme-linked Immunosorbent Assay.
