# Supplementary figures and images for: Thrombosis and antiphospholipid antibodies in Japanese COVID-19: based on propensity score matching
Source: Front Immunol. 2023 Oct 16;14:1227547. doi: 10.3389/fimmu.2023.1227547 (PMC10614020; doi:10.3389/fimmu.2023.1227547)

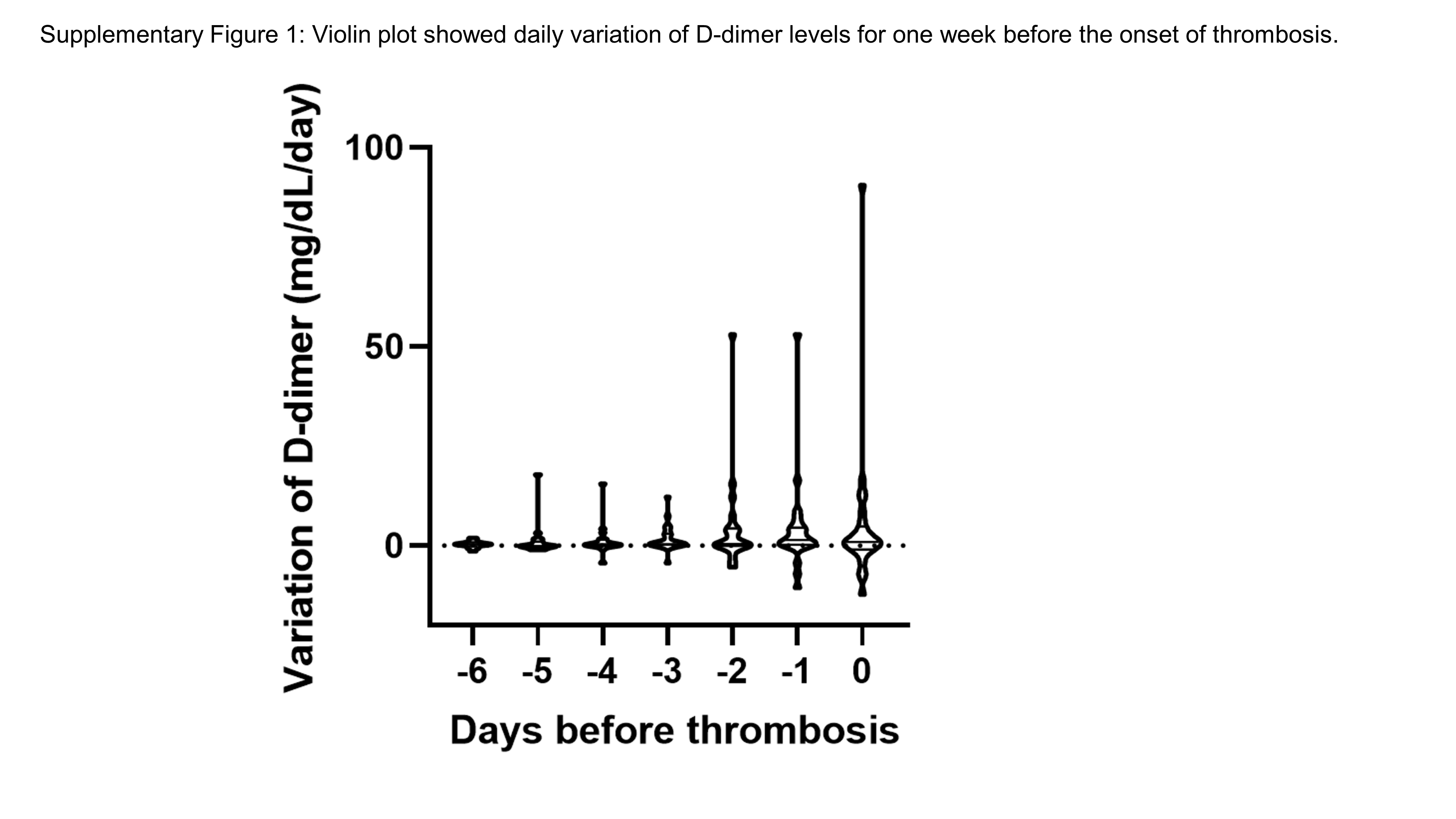

Supplement: Supplementary Figure 1 — Violin plot showed daily variation of D-dimer levels for one week before the onset of thrombosis. [file Image_1.tif]

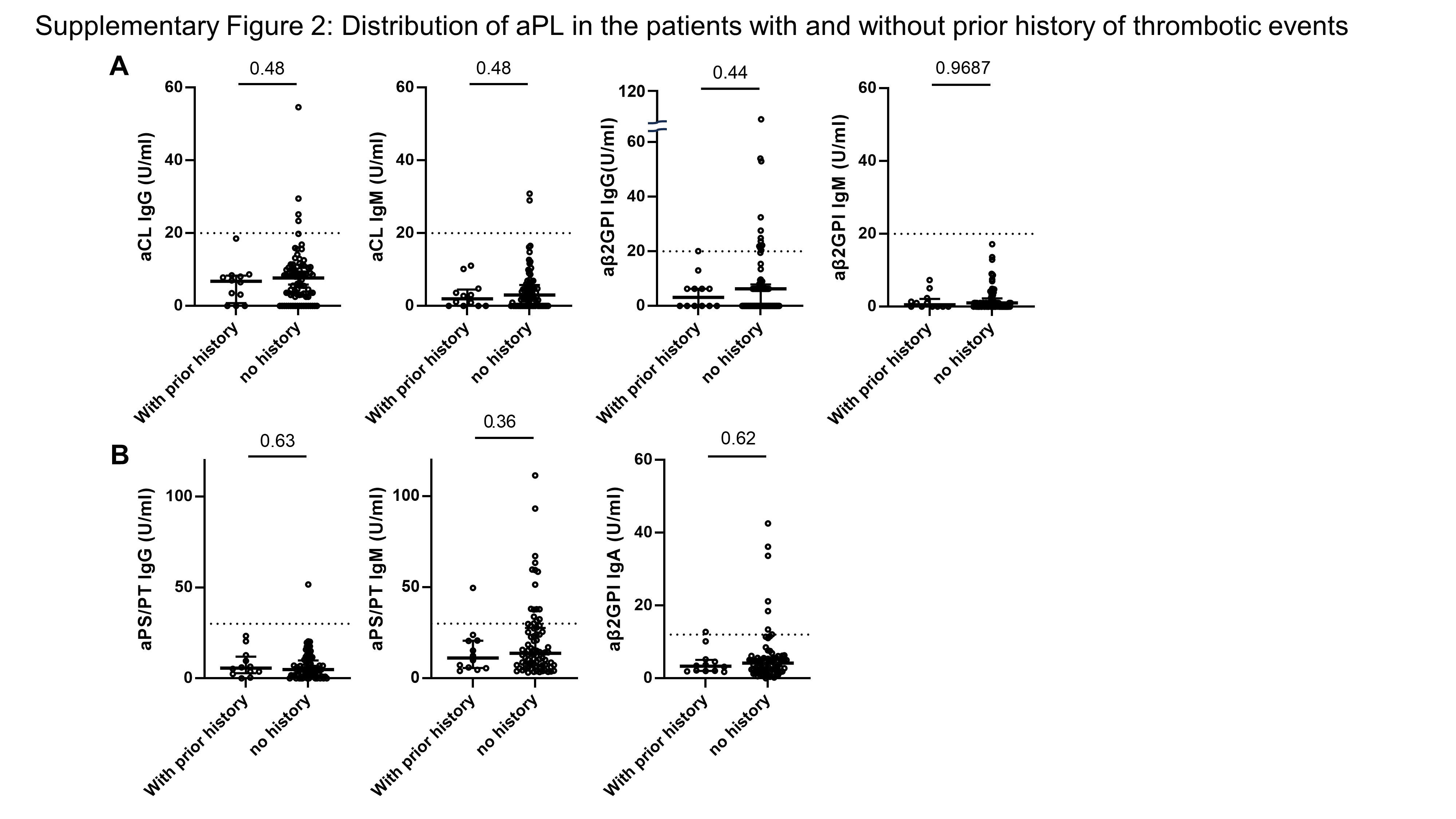

Supplement: Supplementary Figure 2 — Distribution of aPL in COVID-19 patients with and without prior history of thrombotic events. Titers of classic aPL (A) (anti-cardiolipin (aCL) IgG/IgM, anti-beta-2glycoprotein I (aβ2GPI) IgG/IgM) detected by a chemiluminescence analyzer, and titers of non-criteria aPL (B) (aβ2GPI IgA and anti-phosphatidylserine/prothrombin (aPS/PT) IgG/IgM) detected by ELISA in COVID-19 patients with (n=12) and without prior history of thrombotic events (n=82). Values are expressed as median levels [first and third quartile]. Broken lines represent the manufacturer’s cutoff for positivity (20 U/ml for classic aPL, 30 U/ml for aPS/PT IgG/IgM, and 12 U/ml for aβ2GPI IgA). Groups were analyzed by Mann-Whitney U-test. [file Image_2.tif]

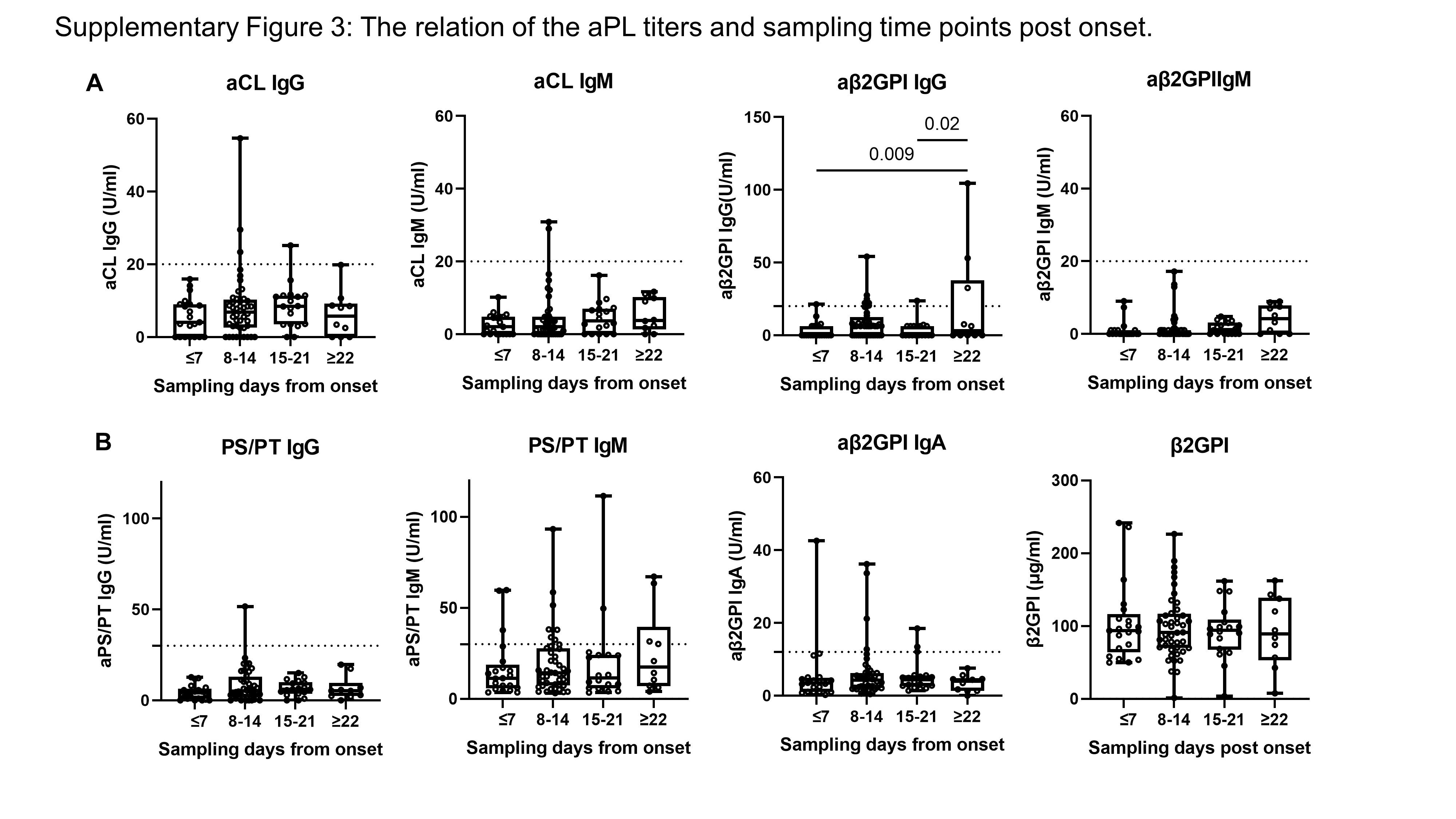

Supplement: Supplementary Figure 3 — The relation of the aPL titers and sampling time points post onset. The samples were categorized into four groups based on the collected days of post onset (DPO). The groups consisted of the samples collected within a week, between 7-14 days, between 15-21 days, and more than 22 days, and the numbers of samples were 21, 44, 18, and 10, respectively. Values are expressed as box-and-whisker plot. Groups were analyzed by one-way ANOVA followed by Tukey-Kramer post hoc test. We showed statistically significant adjusted p-values (overall alpha = 0.05). [file Image_3.tif]

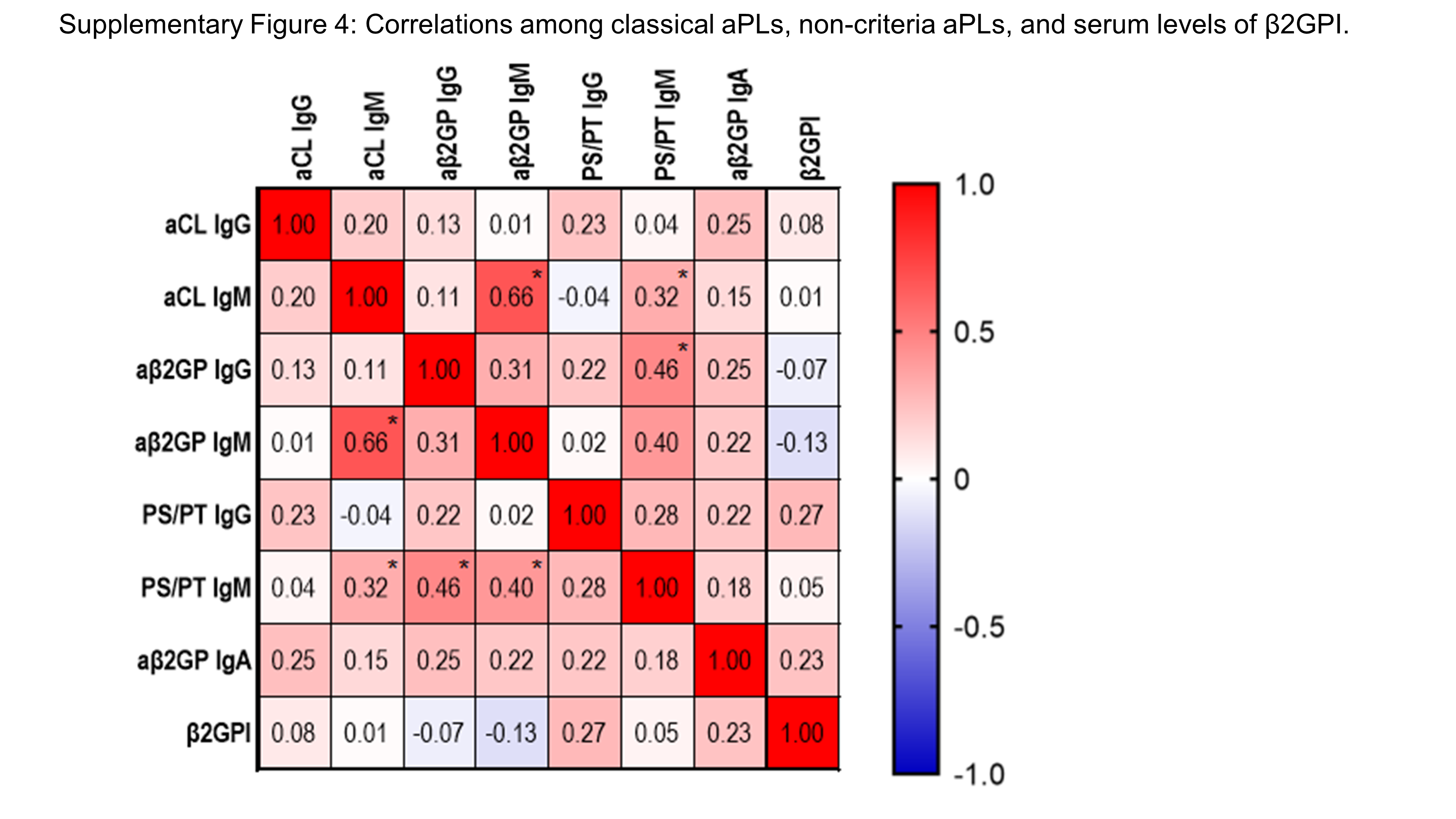

Supplement: Supplementary Figure 4 — Correlations among classical aPLs, non-criteria aPLs, and serum levels of β2GPI. Considering the multiple comparisons, we used p< 0.0018(0.05/28) as statistically significant and added asterisks (*). [file Image_4.tif]
